# Time to start taking time seriously: how to investigate unexpected biological rhythms within infectious disease research

**DOI:** 10.1098/rstb.2023.0336

**Published:** 2025-01-23

**Authors:** Rachel S. Edgar, Aidan J. O'Donnell, Alan Xiaodong Zhuang, Sarah E. Reece

**Affiliations:** ^1^ Department of Infectious Disease, Imperial College London, London SW7 2AZ, UK; ^2^ Francis Crick Institute, 1 Midland Road, London NW1 1AT, UK; ^3^ Institute of Ecology and Evolution, School of Biological Sciences, University of Edinburgh, Edinburgh EH9 3FL, UK; ^4^ 4. Division of Infection and Immunity, Institute of Immunity and Transplantation, University College London, London WC1E 6BT, UK

**Keywords:** circadian clock, rhythms, host–parasite interaction, seasonal, fitness, immune response

## Abstract

The discovery of rhythmicity in host and pathogen activities dates back to the Hippocratic era, but the causes and consequences of these biological rhythms have remained poorly understood. Rhythms in infection phenotypes or traits are observed across taxonomically diverse hosts and pathogens, suggesting general evolutionary principles. Understanding these principles may enable rhythms to be leveraged in manners that improve drug and vaccine efficacy or disrupt pathogen timekeeping to reduce virulence and transmission. Explaining and exploiting rhythms in infections require an integrative and multidisciplinary approach, which is a hallmark of research within chronobiology. Many researchers are welcomed into chronobiology from other fields after observing an unexpected rhythm or time-of-day effect in their data. Such findings can launch a rich new research topic, but engaging with the concepts, approaches and dogma in a new discipline can be daunting. Fortunately, chronobiology has well-developed frameworks for interrogating rhythms that can be readily applied in novel contexts. Here, we provide a ‘how to’ guide for exploring unexpected daily rhythms in infectious disease research. We outline how to establish: whether the rhythm is circadian, to what extent the host and pathogen are responsible, the relevance for host–pathogen interactions, and how to explore therapeutic potential.

This article is part of the Theo Murphy meeting issue ‘Circadian rhythms in infection and immunity’.

## 1. It is about time to uncover the importance of biological rhythms in infectious disease

**Figure 1.**
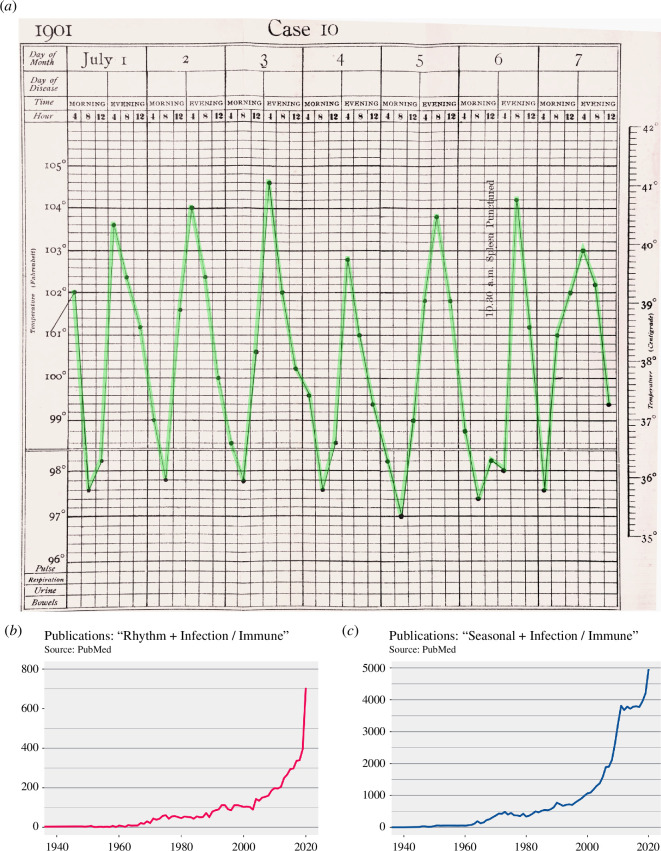
Historical and recent medical and scientific relevance. (*a*) Daily temperature rhythm data from a malaria-infected patient in 1901, collected as part of symptom monitoring to assess antimalarial treatment effects [1] . Upsurge in publications addressing (*b*) circadian/daily and (*c*) seasonal rhythms in infection and immunity, highlighting that seasonal rhythms are more often studied. Graphs depict the numbers of studies published each year from 1940 to 2020 (Source: PubMed search, https://pubmed.ncbi.nlm.nih.gov).

Biological time is a frequently neglected variable in research, but there is growing recognition of its importance in interactions between hosts, vectors and agents of infectious disease. Historic efforts to understand rhythms in infectious disease were often labour-intensive case studies on individual patients (figure 1*a*). In the modern era, rhythmic patterns are emerging from advances in ‘omics technologies, real-time imaging platforms and data science (e.g. [2–5]). Their decreasing cost makes round-the-clock examination of infection dynamics more accessible across all levels of biological scale, from molecular to population studies, from the lab to the field. Consequently, reports of temporal phenotypes during infections are increasingly appearing in the literature (figure 1*b*,*c*). We note that research on ‘seasonal’ infection and immunity far exceeds that on other kinds of ‘rhythms’. However, seasonal studies tend not to harness chronobiology concepts, which is a missed opportunity to apply this robust framework for exploring oscillatory behaviour that we introduce below.

**Figure 2.**
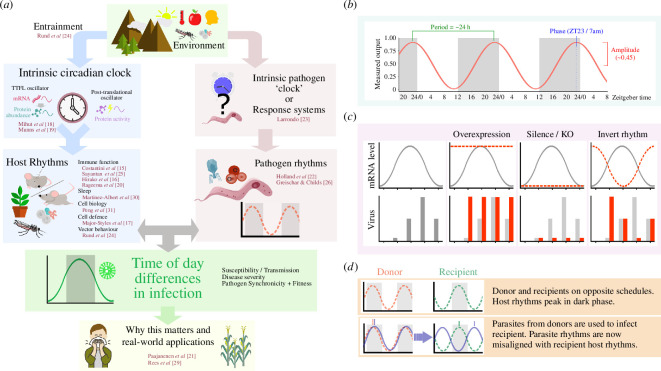
Schematic of methodologies used to interrogate rhythms in infections. (*a*) Environmental factors and host–pathogen rhythms explored in this issue: environmental factors influence the intrinsic circadian clocks of hosts or/and pathogens and response systems to the pathogen. These factors regulate rhythmic activities in both host and pathogen (e.g. immune responses, pathogen replication), affecting infection dynamics. Understanding these interactions is critical in the field of chronopathology, which investigates how rhythms in hosts and pathogens impact disease outcomes and treatment effectiveness. (*b*) Defining rhythmicity: a biological rhythm is characterized by period, phase and amplitude (see table A1 for definitions). Alterations in pathogen rhythms relative to host rhythms can influence infection outcomes over a 24-h cycle. (*c*) Manipulating rhythms: altering host or pathogen rhythms through methods such as overexpression, gene silencing/knockout (KO) or rhythm inversion can impact pathogen activities (illustrated by mRNA level and viral load; natural rhythm and its impacts in grey, perturbations and consequences in red). (*d*) Circadian (mis)alignment: differences in circadian alignment between pathogen and recipient hosts can reveal characteristics of pathogen rhythms and their consequences.

Parasites, viruses, bacteria and other microbes (henceforward referred to as pathogens) encounter host environments that change with the time-of- day or season. For example, circadian rhythms have been characterized across the immune systems of diverse taxa (e.g. plants, mammals, insects [6–12]). In parallel, rhythms have been identified in pathogen traits that underpin their virulence and transmission, including metabolism, replication and dissemination throughout tissues [13–15]. Understanding how rhythms shape interactions between hosts, pathogens and vectors, and why pathogens express their own rhythms, is challenging because it requires disentangling oscillations across multiple interacting species in the context of complex pathogen lifecycles (figure 2).

The original research and perspectives in this theme issue build upon this rapidly developing field of ‘chronopathology' (chronos = time, pathology = study of disease) and highlight future areas of enquiry (figure 2*a*). Mechanisms governing daily regulation of host immunity, metabolism and homeostasis are beginning to be elucidated (e.g. Costantini *et al*. [16]; Hirako *et al*. [17]; Major-Styles *et al*. [18]; Mihut *et al*. [19]; Munns *et al*. [20]; Rageema *et al*. [21]), with circadian clocks often determining the magnitude of responses (Paajanen *et al*. [22]), but why these rhythms exist and their relevance during infection is poorly understood (e.g. Holland *et al*. [23]; Larrondo [24]). Beyond daily timekeeping, hosts and vectors can exhibit seasonal changes in their activity and responses that could affect disease transmission and population dynamics of infections (e.g. Rund *et al*. [25]; Sayantan *et al*. [26]). For pathogens, establishing what drives rhythmic traits, how these rhythms are controlled and how they interact with host timekeeping to impact disease severity and pathogen fitness are major questions (e.g. Greischar and Childs [27]; Hirako *et al*. [17]; Holland *et al*. [23]; Larrondo [24]). Given that the time-of-day of infection can dictate life-or-death outcomes for hosts and pathogens [28,29], answers to these questions are urgently needed. Moreover, understanding the roles of rhythms in infections can inform the development new interventions and the time-of-day to deploy them most effectively.

Chronobiology research has generally focused on elucidating timekeeping mechanisms in model experimental species, most often the house mouse *Mus musculus*, vinegar fly *Drosophila melanogaster*, flowering plant *Arabidopsis thaliana*, fungus *Neurospora crassa* and cyanobacterium *Synechococcus elongatus*, with the molecular fundamentals of endogenous clocks still debated (e.g. Larrondo [24]; Mihut *et al*. [19]). However, chronobiology is also being applied to tackle local and global threats to human health and the environment (e.g. Rees *et al*. [30]; Paajanen *et al*. [22]). Articles in this issue, for example, address how sleep and its timing corelate with infection susceptibility in humans (Martinez-Albert *et al*. [31]), the timing of flight in the malaria mosquito vector *Anopheles stephensi* (Rund *et al*. [25]) and rhythmic activity of *Prochlorococcus marinus*, a marine cyanobacterium that constitutes the largest carbon sink on Earth (Peng *et al*. [32]), as well as explaining the rhythms of pathogens (Hirako *et al*. [17]; Holland *et al*. [23]). Expanding our understanding beyond model organisms will not only aid these efforts, but also uncover fundamental aspects of circadian clock evolution and help identify novel rhythms in microbes of clinical and environmental importance (Larrondo [24]).

While chronobiologists set out to test for the presence of rhythms in hosts and pathogens, or in their interactions (e.g. [33–36]), other studies make these observations by a more serendipitous route. For example, the observation that hepatitis C virus (HCV)-infected patients undergoing liver transplantation experienced faster viral replication when transplant surgery occurred in the morning compared with in the afternoon launched the study of how host rhythms regulate flaviviruses [37]. In addition to discoveries stemming from the clinic, time-of-day effects emerge when researchers alter the schedule of frequently conducted experiments. For example, they may notice that a disease phenotype is more severe when hosts are infected at a certain time- of-day, or that unexpected results stem from sampling at a different time-of-day (e.g. [38,39]). Alternatively, so-called ‘clock genes’ may stand out as important interacting partners for pathogen proteins during infection (e.g. [40,41]).

**Figure 3.**
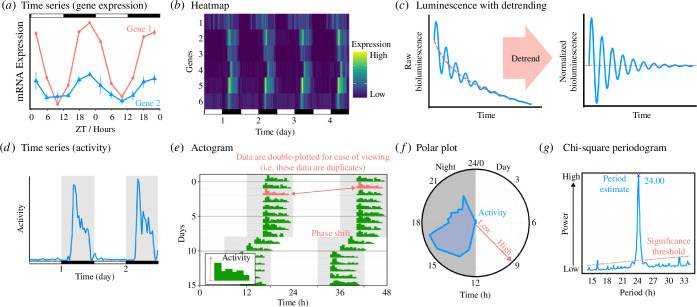
Visualization of rhythms. Appropriate choice of figures depends on the type of data, how it has been processed and the biological insight being illustrated. Indicating when data are collected from day (light) and night (dark) is usually denoted by white-black bars (*a,b,d*), or with grey shaded regions to indicate dark (*d–f*). It is good practice to include raw data (*a*) and this format aids comparison of the phase and amplitude of different groups, but for visualizing many groups, a heatmap is useful (*b*) although while rhythmicity *per se* is visible, resolution of phase and amplitude may be reduced. The intensity and amplitude of measurements from cell-reporters can decay over time, requiring detrending and normalizing to deconvolve the biological rhythm of interest from other shorter- and/or longer-term trends (*c*). When data are from higher resolution time series than in (*a*), the raw traces are often illustrated to give an indication of noise in the dataset (*d*). Behaviour data are commonly plotted as an actogram (*e*), where each row is a different day (i.e. circadian cycle) and double plotting allows each cycle to be compared with the next, with the height of bars indicating activity level. Actograms are particularly useful for illustrating the effects of perturbating an environmental rhythm, which is indicated by moving the white and grey shading between rows (here, a phase shift of 6 h which advances the rhythm is illustrated). Data illustrated in (*c–e*) represent continuous or repeated measures on an individual independent replicate (e.g. a single organism or culture) and can confer higher temporal resolution, less variance and more power to analyses than data represented in (*a,b*), which are often derived from destructive sampling so that each time point represents different replicates. Polar or Rayleigh plots (*f*) are particularly good for visualizing phase because the cyclical nature is clear. In addition to displaying the rhythm and its characteristics, it is good practice to give an indication of the strength of support for the rhythm, so periodograms (*g*) are often included in electronic supplementary figures.

Here, we guide researchers taking forward hypotheses or observations of temporal phenotypes during infections. Our focus is circadian clocks but the tools and concepts extend to biological rhythms with seasonal, infradian (>24 h) and ultradian (<24 h) durations. Engaging with the core concepts and approaches in a new discipline can be daunting, especially when changing gears from conducting lab work at a single biological time- of-day to round-the-clock studies. The substantially increased cost—both to grant budgets and to the sanity of researchers—requires robust identification of key questions and rigorous experimental design. We wish to equip researchers with the knowledge, conceptual framework and confidence to take their research into a new paradigm while avoiding common chronobiology pitfalls. Our ‘hitchhiker’s’ guide aims to: (i) explain why rhythms in infections matter, and what may be gained from considering time-of-day; introduce core chronobiology concepts and how these translate to infectious diseases; (iii) outline different approaches to study rhythms in infections and their application to diverse study systems; and (iv) explore how explaining rhythms in infections can help solve real-world problems. Throughout, we illustrate experimental (figure 2*b–d*) and analytical (figure 3) aspects of working on rhythms, and our glossary (table 1 in appendix A) provides a practical guide that in addition to defining terms, highlights common misconceptions and how to avoid them. While we focus on infectious diseases, our template applies to any study system or topic meeting chronobiology for the first time.

## 2. Conceptual toolkit

Circadian clocks coordinate organisms’ activities with daily environmental rhythms, enabling them to cope with the predictable consequences of the Earth’s solar rotation. Thus, life in a rhythmic world dictates the timing of activities for many organisms. This includes pathogens, for which the environments within their hosts and vectors change dramatically over 24 h, in fundamental cellular processes (e.g. transcription, translation, bioenergetics), systemic physiology and homeostasis (e.g. metabolism, body temperature, immune responses) and whole-organism behaviours (e.g. locomotor activity, reproduction, foraging). These myriad daily rhythms of hosts and vectors offer opportunities for pathogens to exploit and they present dangers to evade at certain times of day [13,15,29,42]. *Vice versa*, hosts and vectors can deploy their rhythms, or may need to overrule certain rhythms, to evade and defend against pathogens [43]. Given that hosts (including vectors) become infected at specific times of day—often driven by rhythms in foraging—and that rhythms govern infection progression, researchers may wish to elucidate:

- What genetic and molecular mechanisms allow host circadian clocks to influence responses to infection? Is it beneficial to fine-tune this control in response to different pathogens?
- Do pathogens possess circadian oscillators of their own and what drives them? Or are they intrinsically arrhythmic and allow host rhythms to enforce rhythmicity—for example, by killing mis-timed pathogens or reliance on the host’s rhythmic cellular machinery for replication and dissemination?
- What are the opportunities and dangers that host rhythms present to parasites and how do pathogens exploit/evade them, respectively?
- Can pathogens determine the time-of-day when they infect and manipulate host circadian rhythms to their own ends? Do host and pathogen rhythms coevolve?
- Is there feedback in which circadian rhythms and immune responses both influence each other? Do they interact to balance resistance against pathogens and tolerance to the costs of infection, and to aid recovery?

Answering these questions requires a framework grounded in well-verified chronobiology concepts. For example, a phenotype that depends on time- of-day does not necessarily constitute a circadian rhythm or reveal an endogenous oscillator, but this is a plausible place to start given that circadian timekeeping permeates biology and was likely an emergent facet of cells (table 1 in appendix A).

### (a) What are circadian rhythms?

Circadian rhythms are approximately 24 h oscillations in behaviour, physiology and cellular activity that are exhibited by most organisms. They rely on intrinsic timing mechanisms and are distinguished from passive responses to environmental fluctuations by three primary characteristics [44,45]. First, circadian rhythms are self-sustaining and persist in constant conditions when no external timing cues are received. Second, circadian rhythms can synchronize their phase to external timing cues (‘zeitgebers’), such as daily light/dark and feed/fast cycles. This entrainment locks the intrinsic oscillation to exactly 24 h and ensures that the rhythmic phenomenon occurs at the appropriate time-of-day, often allowing biological oscillations to functionally anticipate 24 h environmental cycles. Finally, the speed of the rhythm—the period—does not vary over biologically relevant temperature ranges, ensuring that timekeeping is compensated against temperature fluctuations. By contrast, daily rhythms can be generated by a direct response to cyclical environmental stimuli, but these are not anticipatory or endogenous so the same response is mounted independently of the time-of day at which the stimuli occurs. Organization of circadian rhythms, their oscillatory parameters (figure 2*b*) and how they temporally regulate, or ‘gate’, cellular physiology are discussed in detail in this theme issue (e.g. Mihut *et al*. [19]; Paajanen *et al*. [22]).

Daily oscillations are observed at all levels of biological scale, but circadian rhythms are a cellular phenomenon: cells have an endogenous molecular clock that allows them to keep track of time. The canonical model involves delayed transcription–translation feedback loops (TTFL), whereby transcriptional activators drive expression of so called ‘clock genes’ that encode products that directly or indirectly repress their own transcription to yield a 24 h oscillation, which facilitates rhythmic expression of ‘clock-controlled genes’ (CCG). The clock gene circuitry has been characterized in every eukaryotic lineage but is poorly conserved. Thus, absence of canonical TTFL or clock genes in a pathogen that displays a rhythmic phenotype cannot be interpreted as evidence for the absence of a circadian oscillator.

Mammalian circadian clock protein complexes of the TTFL function in concert with global histone modification and chromatin remodelling to generate rhythmic gene transcription, with *ca* 40% of protein-encoding transcripts oscillating in at least one tissue in mice and humans. However, there is a disparity between rhythmic transcripts and rhythms in protein abundance, with often little correlation between the two [5,46–48]. This challenges the linear narrative that TTFL-driven mRNA oscillations lead to rhythms in protein abundance and activity that underpin circadian regulation of cell physiology. Most protein activity is governed post-translationally, and there is growing recognition of the important role that highly conserved kinases and phosphatases play in driving circadian rhythms (e.g. CK1d/e, GSK3a/b and PP1) [19]. In addition, circadian rhythms in post-translational modifications, ion flux, metabolism and protein synthesis occur in many models where the TTFL is non-functional [3,49–53]; O-], suggesting that entirely post-translational molecular timekeeping is feasible in eukaryotes, as demonstrated for cyanobacteria and the FRQ-less oscillator in *Neurospora* [54,55]. Although a more complex picture of the circadian clock is emerging, the output remains the same: most fundamental cellular processes robustly oscillate over the course of 24 h, under laboratory and real-world conditions.

The take-home message is that time-of-day differences in host or pathogen phenotypes may be driven by endogenous biological timekeeping in one, several, or none of these parties. In addition, circadian rhythms at the cellular, systemic or behavioural level of the host may all contribute to driving a time-of-day dependent pathogen phenotype. Discriminating between all these possibilities is not a trivial undertaking, but knowing which rhythms matter and how they are generated is key to whether they can be targeted to improve host health or reduce pathogen fitness.

### (b) Why possess a circadian clock?

Aligning activities appropriately to daily environmental cycles of light/dark, temperature, humidity and UV exposure is an intuitive way to maximize survival and reproduction (i.e. Darwinian fitness) [56]. For example, undertaking risky foraging behaviour when most predators are resting provides a form of ‘extrinsic adaptive value’. Such extrinsic benefits arise from alignment to environmental rhythms, which may be abiotic or belong to other organisms. Organisms may also garner intrinsic benefits from temporally compartmentalizing incompatible internal processes [57]. A classical example of this is DNA replication being undertaken at the time-of-day that minimizes damage from metabolism-induced oxidative stress or UV exposure. In the context of intrinsic benefits, the time-of-day that activities are undertaken does not matter *per se*; having a clock whose time is set by an external rhythm provides a convenient and accurate way to organize internal processes. Furthermore, owing to their self-sustaining nature, circadian clocks confer the ability to anticipate and therefore, prepare for, environmental change. For instance, plants modulate the degradation rate of starch during the night so it is optimally depleted according to when they anticipate dawn and the onset of photosynthesis to replenish reserves [53,58].

Despite the ubiquity of circadian clocks across diverse taxa and their assumed fitness benefits, chronobiology has focused on uncovering the molecular mechanisms driving rhythms rather than their ecological and evolutionary explanations. However, the fitness benefits of biological rhythms have been demonstrated for cyanobacteria, malaria parasites, mice, insects and *Arabidopsis* [34,59–63]. The most commonly used experimental paradigm is to compare proxies for fitness (e.g. replication rate, survival, growth, competitive ability or reproduction) between individuals raised in an environmental cycle that matches their endogenous clock, revealing a competitive advantage over individuals whose clock does not resonate with the duration (period) or timing (phase) of the environmental rhythm (figure 2*b*). For example, in a semi-wild release study, mutant mice with a shorter (<24 h) circadian period length had lower survival and fecundity compared with those with near 24 h periods, and consequently the prevalence of the ‘short-clock’ gene mutation in the population dropped from *ca 5*0% to 20% within 14 months [61]. Further support for the importance of resonating with environmental rhythms comes from cyanobacteria: long-term evolution in a non-rhythmic environment (constant light) favours round-the-clock photosynthesis and metabolism, which is achieved by mutations that break circadian clockwork [64]. However, while intuitive ideas about the biological function (i.e. fitness costs/benefits) of rhythms in various host and pathogen traits are easy to construct, natural selection is complex and often counter-intuitive, and hard to test in rigorous manner [28,29].

## 3. Choose your own temporal adventure

This theme issue demonstrates the breadth of methodology and experimental systems used to investigate rhythms during health and disease (figure 2*a*). It would take several volumes to comprehensively cover all chronobiological tools and techniques used in *in vitro* and *in vivo* lab experiments, field studies, computational models and evolutionary theory. Instead, we present a series of case studies that proceed from different starting points.

### (a) A screen has identified a ‘clock gene’. Is the infection model circadian-regulated?

Many researchers consider timekeeping upon encountering ‘clock genes’, designated owing to their involvement in the transcriptional–translational feedback loops that regulate some rhythmic gene expression. Perhaps a screen yielded direct interaction between a pathogen and a clock gene product, or revealed differences in their abundance, post-translational modification or rhythmic parameters (amplitude, period, phase; figure 2*b*). Clock gene products are transcriptional activators, for example CLOCK and BMAL1 in mammals, or repressors such as the PERIOD and CRYPTOCHROME proteins. These proteins are often promiscuous binders with additional functions beyond circadian or transcriptional regulation [18–20] , so may affect the pathogen or host response in a ‘non-circadian’ capacity. Alternatively, the pathogen may specifically utilize a clock protein during replication to harness or manipulate host rhythms to their own end, including replication, as proposed for CLOCK/BMAL1 and herpesvirus interactions [33,40,65]. Elucidating the molecular interactions between a pathogen and circadian clockwork can be informed by asking the following questions.

#### (i) Are there measurable differences in infection processes depending on the time-of-day of infection?

It is essential to examine this question over several biological scales. For instance, pathogen replication may vary, with specific times of day promoting more aggressive or restrained replication. Furthermore, the time-of-day of infection could influence both local and systemic host responses, including the production of cytokines, the activation of immune cells and the broader inflammatory response. These differences can have knock-on consequences for disease severity, host vulnerability and recovery. Finally, the time-of-day of infection can affect pathogen transmission potential by influencing factors such as dissemination, host behaviour and interactions with other hosts or vectors (e.g. [33,66,67]).

To differentiate between diel and circadian drivers, experiments can be undertaken in constant conditions to infect hosts at different circadian times of day (table 1 in appendix A), while also controlling for any pathogen rhythms (figure 2).

#### (ii) Does genetic or pharmacological disruption of clock protein function impact upon infection?

Exploring the impact of genetic or pharmacological disruption of circadian clock components involves several considerations. Comparing the effects of conditional versus constitutive knockouts of circadian clock genes can reveal how these genetic alterations influence the immune response, pathogen replication and disease severity. The effects of acute reduction of clock protein function—achieved through methods such as siRNA or CRISPR/Cas9-mediated knockdown—should be contrasted with the consequences of stabilizing these proteins, for example, through pharmacological inhibitors or overexpression. Furthermore, investigating how post-translational modifications of clock proteins, such as phosphorylation or ubiquitination, affect their activity and consequently the host response to infection can provide insights into the dynamic temporal regulation of these proteins. Alterations in these modifications, potentially induced by pharmacological agents, could significantly alter the course of infection. Ultimately, if a clock gene product is critical to an infection process, testing this with a targeted therapeutic may seem enticing. The challenges and limitations of this approach, including how to probe for a *bona fide* circadian impact by avoiding off-target effects and identifying specific mechanisms of action, are discussed in detail in this issue [20].

#### (iii) Does infection alter host rhythms?

Understanding whether infection alters host circadian rhythms requires analysis at multiple scales, covering the molecular, physiological and behavioural levels [68]. At the molecular level, infection may disrupt the expression of core clock genes and proteins, which can be monitored using techniques such as bioluminescence assays, or by transcriptomic and proteomic studies of samples collected from circadian-entrained cells or animals [41,69,70]. At the physiological level, infection might influence circadian-regulated processes such as hormone secretion (e.g. cortisol, melatonin), body temperature or metabolic activities, either directly or as a secondary consequence of the immune response [70–72]. Infection may alter circadian-regulated behaviours, such as sleep–wake cycles, feeding patterns or general activity levels, with changes potentially observable through activity monitoring [73,74]. These disruptions across multiple levels would indicate a significant interaction between infection and the host circadian system.

### (b) A rhythmic host process impacts my pathogen. Which host rhythm(s) is important?

Host immune responses to infection at the cellular, tissue and systemic levels are often gated by circadian clocks. Intuition suggests that because immune responses defend against pathogens, clock control of immune responses must cause pathogens to be killed in a time-of-day specific manner. Indeed, anti-infection defences are thought to peak during the host’s active phase because this is when pathogens (especially if food-borne) are most likely to be acquired. However, whether immune rhythms are particularly important for standing defences is poorly understood and other aspects of host rhythms might have more impact than foraging on infection risk. For example, some of the deadliest infections are vector-borne and acquired in the host’s rest phase and respiratory viruses are acquired when hosts aggregate, which can be in either the rest or active phase, depending on when the host species has most social interactions. Mechanisms governing rhythms in immune responses and the consequences of these rhythms are relatively well understood, especially for components of the innate response and non-infectious diseases [6,8,10–12,21,43]. Asking how immune rhythms affect infection processes requires considering to what extent an immune factor is regulated in a rhythmic manner to prevent immunopathology, be energetically efficient or for other intrinsic benefits such as temporally compartmentalizing repair and pathogen attack.

Pathogens also rely on their hosts for resources required to replicate and, in the case of viruses, the machinery to synthesize their proteins and assemble new particles for onwards transmission. Given the proportion of metabolites and proteins with differential abundance and activity over 24 h and the myriad cellular processes gated or licenced by the circadian clock [2,19,22], it is likely that a critical resource, protein or process is more accessible at one time or another over 24 h [14,17]. In some scenarios, for example the rhythmic expression of proteins on the cell surface that enable virus binding and entry, maxima and minima of the rhythm substantially alter infection outcomes [75,76]. In other cases, while a particular process may be limiting at certain times of day (especially during sickness), pathogens can reprogramme cells to promote replication, with hosts responding to limit resources and pathogen dissemination [15,33,76]. In these more nuanced instances, any initial time-of-day difference may amplify or diminish as infection progresses. Simultaneously monitoring host rhythms and infection progression presents a challenge because both are dynamic processes.

Ultimately, to establish whether a host rhythm underpins the time-of-day effect on a pathogen, the important questions to answer are the same as for §3a. Altering expression of the rhythmic protein, availability of the rhythmic metabolite or activity of the rhythmic process only tells you whether its presence required for infection, not whether *its rhythmicity* is required. To demonstrate this, the rhythm must be ‘flatlined’ or inverted (figure 2*c*), or host and pathogen rhythms misaligned (figure 2*d*).

### (c) A pathogen exhibits a rhythmic phenotype. Does it have a clock?

Do not assume that time-of-day dependent phenotype is the result of a genetically encoded, endogenous pathogen clock because environmental and host factors can drive pathogen rhythms, discussed previously in §3a. The general TTFL structure is found across the kingdoms of life but the molecular components are not conserved across taxa, whereas post-translational regulatory processes and kinase/phosphatase involvement are conserved, so all is not lost in the absence of defined canonical ‘clock genes’. The challenges of identifying timekeeping mechanisms *de novo* in non-model organisms—including insights, pitfalls and caveats—are discussed in detail in this issue [24]. Establishing the existence of an independent endogenous circadian clock is especially difficult for non-model pathogens [77,78] and appropriate approaches depend on the following:

- Can the pathogen survive outside the host in constant environmental conditions for a long enough period to observe rhythmicity (ideally >72 h)?
- What environmental cue(s) synchronize or entrain the pathogen’s rhythms?
- What can be measured to gauge rhythmicity and for intracellular pathogens, would this output from a putative clock function outside the cellular environment?

How questions (i) and (ii) are tackled depends on the biology of the focal pathogen, but for ideas see [77–80]. Furthermore, several robust and independent oscillatory outputs may be required (e.g. transcriptional, metabolic, post-translational).

Whether this avenue is worth pursuing will depend on how important it is to establish the existence of an endogenous, self-sustaining and temperature-compensated oscillator in the pathogen: trying to conclusively prove the converse—that there is no clock—is extremely challenging. Furthermore, some oscillators, especially if evolutionarily ancient, may only fulfil a subset of criteria. Instead, ask why you need to determine the mechanism of pathogen rhythmicity and whether it is self-sustaining or an environmental response; it is still encoded by pathogen genes— and targetable by medical interventions.

### (d) A pathogen exhibits a rhythmic phenotype. Why has this evolved?

While pathogens are sheltered from rhythms in the abiotic environment when living inside others, they are exposed to biotic rhythms belonging to their host/vector. Rhythms in the biotic environment dictate the time-of-day when nutrients (e.g. when food is acquired) and other resources (e.g. ligands used for invasion, replication machinery) are available to pathogens, when dangers are present (e.g. the migration of immune cells) and activity/rest cycles determine when transmission opportunities occur (e.g. the generally nocturnal biting activity of malaria-mosquito vectors [25]). By knowing whether a biotic environmental rhythm presents a danger or an opportunity to a pathogen, one can predict how the pathogen should respond with its own rhythmic strategy (e.g. [23]).

The phase of a pathogen’s rhythm(s) might confer fitness benefits via maximizing the exploitation of host resources or via evading dangers, and pathogens that replicate within the host may also garner fitness benefits from synchronizing their activities with each other [81]. For example, synchronizing waves of replication may enable closely genetically related pathogens to overwhelm host defences, communicate to co-ordinate their developmental decisions or manipulate host rhythms to their own end. However, explaining why a pathogen exhibits a rhythm requires more than an intuitive link between pathogen fitness and an environmental rhythm. This is very challenging when the mechanism driving the pathogen’s rhythm is unknown because perturbating it may not be possible. Assuming it is possible to perturb the pathogen’s rhythm and measure impacts on pathogen fitness (or proxies), asking why a pathogen exhibits a rhythm requires considering the following questions.

#### (i) Why does timing matter?

Ideally, one perturbs the phase of the pathogen’s rhythm(s) relative to the host rhythm hypothesized to determine pathogen fitness and observes costs to the pathogen (e.g. [82]). This is best achieved by exposing a pathogen to its Zeitgeber or time cue in a manner decoupled from the fitness-determining host rhythm to ‘trick’ the pathogen into displaying a suit of different rhythms while the important within-host environmental rhythm is held constant.

#### (ii) Are other aspects of the pathogen’s rhythm important?

That pathogens should undertake activities at the time-of-day that maximizes their fitness is obvious, and so, natural selection is expected to act on the timing (phase, figure 2*a*) of pathogen rhythms. Yet, for pathogens that replicate within the host, the degree of synchrony (measurable via the amplitude) in the activity of individual pathogen cells is also subject to selection. For example, independently of the phase of the pathogen activity, high synchrony could benefit pathogen fitness if collective action facilitates host exploitation. Alternatively, high synchrony in rhythmic replication could be costly if it causes closely (clonally) genetically related pathogens to inadvertently compete for time-limited resources. In cases where extremely high synchrony is costly, pathogens could evolve to be less synchronous via variation in phase and/or period, resulting in different combinations of phase, period and amplitude being equally fit (figure 2). These combinations might be exhibited across individual pathogen cells within an infection (plasticity, see §3d(iv)), or different pathogen genotypes or species might evolve different combinations. Testing these hypotheses is not straightforward because the expansion and contraction of pathogen numbers during infections can cause intuitive measures of synchrony and replication rate to be extremely biased, as explored in detail in this issue (see [27]).

#### (iii) Does the pathogen gain intrinsic benefits of rhythmicity?

If a pathogen maintained in an arrhythmic environment requires a rhythmic time signal to maintain its fitness, this suggests the pathogen uses the within-host environmental rhythm to set the time of compatible (or not) activities. Thus, the host’s rhythm provides convenient timing information rather than being a selective driver of the pathogen’s rhythm.

#### (iv) Does the pathogen’s rhythm alter as infections progress, or in different parts of its lifecycle?

Infections are a set of dynamic processes, which may mean that different within-host environmental rhythms are important at different stages of disease progression (e.g. acute versus chronic stages, in the host or vector) and pathogens actively alter their rhythms to suit. Further complexity arises when pathogens express different rhythms at different points in their lifecycle. For example, while rhythmic replication in the blood of the vertebrate host is thought to maximize the within-host fitness of malaria parasites, it is possible that rhythmic replication is also (or alternatively) phased to align the infectiousness of transmission stages to the nocturnal biting of Anopheline mosquito vectors [14]. This highlights that examining the consequences of a pathogen rhythm in only part of its lifecycle can give a misleading view of its adaptive value and selective drivers, as explored in this issue [23].

Overall, pathogen rhythms may be shaped by selective forces associated with the benefits and costs of aligning with multiple rhythms in their biotic and abiotic environments, the intrinsic benefits of rhythmicity and the potential for phase and synchrony to affect fitness independently. This means a pathogen’s rhythm might not appear optimal with respect to one aspect of its rhythm, or perfectly aligned to a specific host rhythm, because it is a compromise of multiple evolutionary pressures, past and present.

## 4. Analysis toolkit: dealing with time series data

Measuring traits around the clock produces time series datasets in which a repeating ‘circular’ signal(s) is tested for, rather than a single trend across the whole dataset (figure 3*a*). Finding such patterns amongst noise can be difficult so a robust workflow requires a good dataset, establishing whether there is a rhythmic signal, describing the parameters of the rhythm and then, if applicable, comparing those parameters between treatment groups, before displaying findings (data visualization; figure 3). For most purposes, an ideal dataset has regular (every 2–4 h) equidistant datapoints, spanning at least twice the length of the predicted cycle (e.g. 48 h for a circadian rhythm), with independent replicates [83,84]. Collecting time series from the same individuals over multiple circadian cycles is also desirable, such as when additional dynamic variables that have associated rhythms can be measured (e.g. [23]) or generating expensive ‘omics data. Equidistant datapoints are the main priority because many statistical algorithms to assess rhythmicity (such as JTK_CYCLE, ARSER, RAIN [85–87]) fail without these. For reasons of financial costs, operational feasibility and tractability of infections, compromises between the number of time points and replicates often must be made; here equidistant samples across two or more cycles are better than more samples within one cycle [84]. However, for detecting rhythms in situations where the number of pathogens or immune cells changes during sampling, for example, uneven sampling intervals can avoid overestimating amplitude [88,89].

Analysing time series data is at a minimum a two-step process of determining whether a significant rhythmic signal exists before then estimating the parameters of the rhythm (period, amplitude, phase; figure 2*a*). Algorithms to test for rhythmicity are broadly categorized as curve-fitting methods: generating curves that vary in shape and assessing how closely each curve fits the original data (e.g. COSOPT [90], harmonic regression, Fast Fourier Transform Nonlinear Least Squares (FFT-NLLS)), or spectral based methods that extract an entire spectrum of signals in the data and determine which is the strongest (e.g. Lomb–Scargle periodogram, Maximum Entropy Spectral Analysis (MESA)). Other approaches include non-parametric tests (e.g. JTK_CYCLE and RAIN) that are widely used for ‘omics datasets’. Choosing between different methods (with disparate statistical origins) may seem overwhelming but this variety benefits researchers because the strengths and weaknesses of all approaches differ, and it is good practice to use a combination of methods to confirm rhythmicity [91]. For example, spectrum-based methods (e.g. MESA) are excellent at estimating parameters but will do so for any signal in the data, regardless of whether it is biologically or statistically significant. It is important to use these algorithms in conjunction with others that provide a level of confidence that there is an underlying rhythm (harmonic regression, FFT-NLLS, JTK_CYCLE, RAIN).

Analyses can be undertaken using several tools for time series data (e.g. [92], BioDare2, CIRCADA [93], PyBoat, CircaCompare, metacycle, Rethomics ([92]*| Actimetrics*, n.d.; [94], n.d. [11,91,95–97])). For its user-friendliness, we recommend the web-based BioDare2 [91,98]. BioDare2 performs detrending (which helps in determining rhythmicity in data where a pattern might be masked by linear trends such as pathogen replication; figure 3*c*), includes six methods for parameter estimation and provides visualization of the dataset and of parameter estimates. BioDare2 is also an open data repository, facilitating open science and collaboration. The methods implemented in BioDare2 are particularly powerful for estimating period length, which is one of the more difficult parameters to estimate accurately [91]. However, while BioDare2 can analyse any data (e.g. luminescence, gene expression, pathogen density), we recommend companion software such as Clocklab [92] (Actimetrics, USA), or the R package Rethomics [95] for behavioural (e.g. host activity, figure 3*d*) data. Rethomics creates plots traditionally used to visualize activity (e.g. actograms; figure 3*e*) and calculates measurements specific to behavioural data such as the duration and onset of activity bouts. For datasets with very complex dynamics that obscure rhythms, typical of infections, bespoke statistical models or simulations may be necessary [27].

For experiments whose data are solely used to test for, and characterize, a rhythm, no further analysis is necessary. However, for experiments designed to compare rhythms across treatment groups or categories (e.g. figure 3*a*) the next step is to compare the proportion of replicates that are rhythmic and the parameters of those rhythms between groups. Standard statistical methods are deployed, such as generalized linear models (GLMs) with mixed effects if using groups that have non-independence due to repeated measures from replicates. Note that phase is a circular statistic (i.e. the end of a circadian cycle is also the start of the next cycle), necessitating approaches such as Bayesian circular GLMs [99]. Alternatively, all-in-one curve fitting and parameter estimating methods such as CircaCompare and DoDR can be used to compare rhythmicity across groups [11,100].

Regardless of what an experiment was designed to test and its outcomes, data visualization is essential. How rhythms and their parameters are best illustrated (figure 3) depends on the type of data (e.g. an actogram (figure 3*e*) for locomotor activity, or a heatmap (figure 3*b*) for gene expression) and the hypothesis being tested (e.g. change in a rhythm over time, or comparison between treatment groups). Given the circular nature of time-series data, polar plots appropriately display phase and amplitude in an easy-to-understand format similar to a wall clock (except over 24 h instead of 12; figure 3*f*). Periodograms (figure 3*g*) provide valuable information on the strength of a rhythm as well as its period, and often form an essential aspect of supplementary information in publications.

## 5. Translational potential

Circadian rhythms underpin homeostasis and health, and the detrimental consequences of circadian disruption caused by modern lifestyles are well-recognized. Many people will have experienced the negative effects of ‘jet lag’, where the internal phase of their body clocks mismatches with their new time zone. Furthermore, circadian misalignment under controlled laboratory conditions disrupts metabolic homeostasis and immune function in human subjects after only a few days. Prolonged and repeated circadian desynchrony during shift work is associated with severe morbidities including cancer, obesity and type 2 diabetes, and loss of robust circadian timekeeping is a feature of ageing and neurological disorders [101]. In addition to clinical therapeutics, combating disease vectors, crop security and preventing antimicrobial resistance may all benefit from considering time-of-day, as discussed in detail in this theme issue [30].

Protecting host rhythms during infections has great potential to alleviate symptoms for humans as well as other animals. Reinforcing host rhythms may also promote recovery from infection, analogous to the detrimental impact of rhythm disruption on recovery from trauma [102]. Host rhythms might be harnessed to make therapeutic interventions more effective, for example vaccine efficacy may depend on the time-of-day of administration [103]. That >50% of the best-selling drugs in the USA have targets that cycle in a circadian manner highlights the value of at least taking time-of-day into account when treating infections [104,105]. Administering a drug at a particular time to synergize with rhythmic immune responses could enhance efficacy or allow lower doses to be used.

Similarly, pathogens' rhythms affect their fitness, so explaining how their rhythms are controlled and why they are beneficial should stimulate novel approaches for reducing disease severity and transmission. For example, the ability of malaria parasites to slow their replication rhythm confers tolerance to antimalarial drugs [106,107]. Drugs that disrupt pathogen rhythms or take advantage of rhythmic vulnerabilities in pathogen replication may make treatment more effective, especially for drugs with short half lives. These possibilities highlight the need for better translation of chronobiology within and beyond academia, especially with policymakers and stakeholders, as championed by the BioClocksUK community [30].

## 6. Conclusions

Observations that pathogens express rhythmic behaviours date back to the Hippocratic era and experiments in the 1960s showed that disease severity is a function of the time-of-day of infection, yet general explanations for these phenomena are lacking. Our understanding of the roles of rhythms during infection is still in its infancy, but this novel area of biological enquiry is tractable and translatable. Over the past few decades, chronobiology has focused on ‘what makes a clock tick’ so the time is right to disseminate this knowledge into other disciplines. The role of biological timekeeping during infection has been neglected because different aspects of the underlying biology are studied by disciplines (chronobiology, immunology, evolutionary ecology, parasitology, microbiology, plant sciences, medicine) that often progress independently. While the complexities of considering the rhythms of all parties involved in infections brings new challenges to chronobiology, this topic offers a rare opportunity to explain phenomena across scales of biological organization. We hope the expansive science presented this theme issue, and our guide to getting started, will inspire readers to incorporate chronobiology into their research using a multidisciplinary approach.

## Appendix A

See table 1

**Table 1.** Consensus chronobiology terminology with common confusions and contentions.

term	definition	useful notes	misconceptions/clarifications
a**mplitude**	The difference between the peak and the average level of a rhythmic process	amplitude reflects the intensity of a rhythm, or the synchrony of rhythms of cells in a sample	**misconception**: higher amplitude equals a stronger circadian rhythm **clarification**: amplitude indicates the magnitude of temporal variation, but a higher amplitude does not necessarily indicate a healthier or stronger rhythm
**arrhythmic**	no evidence of regularity in the pattern of occurrence.	a pattern might not exhibit 24 h periodicity but might be rhythmic with a much shorter period (e.g. an ultradian rhythm), or a longer duration (e.g. lunar and seasonal rhythms)	**misconception**: arrhythmicity can be concluded from data analysi. **clarification**: confidence in categorizing arrhythmicity depends on the parameters in the analysis, including the range of periods that a cycle can take, the number of harmonics allowed and the *p*-value cut-off
**biological clock**	an internal timekeeping mechanism that generates and regulates the timing of biological rhythms	biological clocks are the systems or mechanisms (often molecular and neural) that generate rhythms	**misconception**: biological clock and biological rhythm are the same **clarification**: a biological clock is the underlying timekeeping mechanism, while a biological rhythm may or may not be the output created by a clock
**biological rhythm**	any repeating physiological or behavioural cycle, not necessarily endogenous.	the observable patterns that clocks produce (e.g. sleep–wake cycle, body temperature fluctuations) that can exist at multiple timescales, not just the 24-h circadian rhythm.	
**central and peripheral clocks**	in mammals the suprachiasmatic nucleus (SCN) is an important pacemaker, contributing to the timing of other (peripheral) clocks within an organism	a hierarchy of clocks—that was applied to mammalian circadian organization—is more complex than previously assumed.	**misconception**: the SCN sets the time for all other clocks within an organism **clarification**: peripheral clocks have more autonomy that previously thought and some bi-directional feedback between the SCN and other clocks occurs
**circadian clock**	a self-sustaining oscillator that allows an organism to exhibit endogenous 24 h rhythms at the molecular, cellular, physiological, behavioural or population levels	the transcription–translation feedback loop (TTFL) is most commonly studied but recent work is revealing that evolutionarily older oscillator mechanisms exist	**misconception**: all circadian clocks use the same mechanism **clarification**: different taxonomic groups use different genes to run their TTFL, and non-transcriptional and post-translational oscillators also exist
**circadian time (CT)**	notation to indicate the internal clock's timekeeping, referring to time-of-day observed in the absence of external cues.	following a switch from 12 : 12 light : dark (LD) conditions to constant dark, CT0−12 represents subjective day (equivalent to night in LD), while CT12−24 represents subjective night (equivalent to day in LD).	**misconception**: CT is synchronized to the 24 h day **clarification**: CT reflects the organism's internal rhythm, which might slightly differ from 24 h
**clock gene**	component of the molecular clock mechanism important for overt circadian rhythm(s), which often feeds back to affect its own expression.	absence or mutation leads to either arrhythmicity or altered overall circadian function; clock genes may also have additional functions beyond circadian regulation	**misconception**: all genes showing circadian patterns are clock genes **clarification**: clock genes are those that directly regulate the circadian rhythm, not just any gene with circadian expression patterns
**clock mutant**	an organism with a mutation in one or more clock genes, leading to altered circadian rhythms	clock mutants help study the role of specific components in circadian rhythms. e.g. full-body Bmal1 knockout mice, lose all circadian rhythms and suffer from health issues, so tissue-specific KO models are often used	**misconception**: all mutations in clock genes result in complete rhythm loss **clarification**: some mutations may only alter the period or phase of the rhythm rather than abolishing it entirely; for clock genes with additional functions, those are also affected
**clock-controlled gene**	a gene whose expression is influenced by the circadian clock but is not part of the clock itself	these genes show circadian patterns of expression but are regulated by clock genes	**misconception**: clock-regulated genes are components of the clock mechanism **clarification**: clock-regulated genes are influenced by the circadian clock but do not form part of its core mechanism; the clock ticks normally without them
**constant conditions**	environmental conditions where an external time cue has been removed, e.g. constant darkness (DD)	these conditions allow the study of an organism's circadian rhythm without external influence; in DD, circadian time CT0−12 corresponds to the subjective day and CT12−24 corresponds to the subjective night	**misconception**: constant conditions dampen rhythms **clarification**: constant conditions reveal the organism's internal rhythms, which continue to persist without external cues
**coupling/ decoupling**	coupling allows the synchronization of circadian rhythms among cellular clocks or between the SCN and peripheral clocks, or enables synchrony between individual cells, or organisms	coupling involves intercellular communication (e.g. via signalling molecules or electrical activity) that ensures alignment of cell rhythms and synchronization; decoupling can occur owing to clock disruptions or pathological states, leading to misaligned rhythms or loss of synchrony	**misconception**: decoupling always indicates a pathological or negative state **clarification**: decoupling can be an adaptive response to changing environmental conditions; not all cellular rhythms are perfectly coupled at all times, and coupling strength can vary across tissues and conditions
**diel**	a rhythm with a 24 h period in the presence of a light–dark cycle	diel is usually used to refer to rhythms that follow 24 h LD cycles, but are not known to be the output of a circadian clock	**misconception**: diel and diurnal share the same definition **clarification**: diel refers to a 24 h pattern; diurnal means active predominantly during the light phase
**diurnal (nocturnal)**	active during the day (or night).	diurnal animals are awake and perform most activities during daylight and nocturnal animals at night; diurnal and nocturnal are extremes at opposite ends of a spectrum with other patterns between	**misconception**: diurnal animals are completely inactive at night **clarification**: diurnal animals may have some level of activity during the night, but it is minimal compared with daytime activity (*vice versa* for nocturnal)
**entrainment**	the process by which external cues (zeitgebers) align the internal circadian clock with the external environment	entrainment primarily occurs through exposure to light, which is the most powerful zeitgeber for mammals. other zeitgebers include food, temperature, and social interactions; entrainment allows the circadian clock to adapt to changes such as seasonal light–dark cycles	**misconception**: entrainment is synonymous with synchronization **clarification**: entrainment specifically involves the process of resetting the internal clock based on external cues, while synchronization can refer to the alignment of internal rhythms with each other or with external cues, which may occur independently of entrainment
**free running**	the natural rhythm observed under constant conditions, reflecting the organism's internal clock	the free-running period is typically close to 24 h but may be shorter or longer depending on the species.	**misconception**: free-running rhythms are unpredictable **clarification**: free-running rhythms are predictable and follow the internal circadian clock, even without external cues
**gating**	the circadian clock restricts biological processes or responses to particular phases in the 24 h cycle, creating time windows of increased sensitivity or activity	zeitgebers can be subject to gating, and gating can occur to responses to both exogenous and endogenous cues or stimuli (e.g. light, hormones, pathogens); gating explains why functions such as DNA repair or immune responses occur more frequently at certain times of day	**misconception**: gating is often mistaken for rhythmicity **clarification**: rhythmicity describes regular cycles, while gating adds a control layer; while gating increases sensitivity at specific times, processes can still happen outside these windows, with reduced efficiency
**jetlag**	a temporary disruption of the circadian rhythm caused by rapid travel across multiple time zones, leading to a misalignment between the internal clock and the new local time	symptoms of jetlag include sleep disturbances, daytime fatigue, cognitive impairments and digestive issues; recovery time varies based on the number of time zones crossed and the direction of travel (eastward versus westward)	**misconception**: jetlag is just feeling tired from travel **clarification**: jetlag involves a comprehensive misalignment of circadian rhythms, affecting multiple physiological processes' eastward travel generally causes more severe jetlag because it requires a phase advance, which is harder to adjust to
**light–dark cycle (LD)**	a 12 h light/12 h dark cycle is most often used to simulate natural day and night. The duration of the light part of the day is referred to as the photoperiod	the LD cycle is used to synchronize the rhythms of experimental subjects; the durations of light and dark can vary; e.g. 18 h light and 6 h dark simulates a summer photoperiod in temperate zones, and non-24-h cycles (T-cycles) occur when the durations of light + dark are not 24 h.	**misconception**: rhythms observed under a LD cycle are always driven by a clock **clarification**: rhythms can be driven by responses to external LD changes, or other environmental signals
**masking**	a phenomenon where an external factor (e.g. unexpected light exposure) directly affects an organism's behaviour/physiology, temporarily concealing one or more circadian rhythm	masking can obscure the true circadian rhythm(s), making it appear different from what the internal clock dictates	**misconception**: masking and entrainment are the same **clarification**: masking prevents the detection of the circadian rhythm, while entrainment is the process of aligning the internal clock to external cues
**onset of activity (or rest)**	the time point when an organism begins its active phase, often used as a marker of the start of the subjective night in nocturnal animals (*vice versa* for rest)	for many nocturnal animals under a 12 : 12 LD cycle, the onset of activity typically occurs around lights off. In constant darkness (DD), this is measured by the circadian time (CT12) (*vice versa* for rest).	**misconception**: onset of activity is always tied to the external light cue **clarification**: by constant darkness, activity onset is driven by the internal circadian clock (*vice versa* for rest)
**phase, phase change/shift**	a specific point in the rhythm cycle, such as the timing of peak activity; phase advance: a shift in the circadian clock to an earlier time (e.g. waking up earlier); phase delay: a shift to a later time (e.g. going to sleep later); phase shift: a general term for any change in the timing of the circadian rhythm	phase depends on external cues like light, or internal factors; phase shifts are part of entrainment and occur naturally (e.g. during adolescence or aging) or owing to external factors (e.g. light exposure, jetlag); phase advances and delays refer to specific types of shifts; for example, a phase advance would mean the rhythm starts earlier than usual, while a phase delay means it starts later	**misconception**: phase shifts only indicate a disorder **clarification**: phase shifts can be normal adaptations or responses to environmental changes, such as adjusting to a new sleep schedule or seasonal changes in daylight or travel across time zones
**period**	the duration of one complete cycle of a rhythmic process, such as the time from one peak of activity to the next	in circadian rhythms, the period is often close to 24 h but can vary slightly depending on species, individual, and environmental conditions	**misconception**: circadian period must be exactly 24 h **clarification**: the circadian period can vary slightly, typically ranging from 23 to 25 h, depending on the species and conditions
**subjective day (or night)**	the phase of the circadian cycle that corresponds to the rest period for nocturnal animals or the active period for diurnal animals, occurring in the absence of light cues (*vice versa* for subjective night)	by constant darkness (DD), the subjective day for nocturnal animals corresponds to CT0−12; for diurnal animals, subjective day is the phase when they would normally be active if light cues were present (*vice versa* for subjective night)	**misconception**: subjective day is the same as actual day **clarification**: subjective day is defined by the organism's internal clock and may not align with the external day, especially under constant conditions (*vice versa* for subjective night)
**synchronization**	the alignment or coordination of an organism's internal biological rhythms, either within the body or with external environmental cues	essential for maintaining overall physiological coherence and health; disruptions in synchronization can lead to various health problems, including metabolic disorders, immune dysfunction and mood disturbances	**misconception**: synchronization is often confused with cell cycle synchronization, which refers to the alignment of cell division phases in a population of cells **clarification**: cell division synchronization may involve timing, but via different mechanisms than circadian synchronization; synchronization should be defined and whether it refers to circadian or cell populations made clear
**temperature compensation**	a mechanism that ensures the circadian clock maintains its stable period over different operating temperatures	the speed of a clock with temperature-sensitive molecular reactions would vary according to the body temperature of its owner; environmental temperature can act as a Zeitgeber	**misconception**: temperature has no impact on circadian timekeeping **clarification**: clocks are only compensated across a biologically relevant temperature range
**time-of-day variation**	a quantity of a biological system differs according to the time-of-day when it is measured	day (light phase) and night (dark phase) are often compared	**misconception**: evidence for a circadian clock **clarification**: clock driven, and non-endogenous rhythms, cause time-of-day variation
**zeitgeber**	‘time giver'; external cue (e.g. light) that aligns biological rhythms via entrainment.	zeitgebers are used to align rhythms to environmental time, like resetting a watch to the correct time. Zeitgeber time (ZT) usually refers to hours since dawn (or lights on)	**misconception**: zeitgebers are only light-related cues **clarification**: other factors such as temperature, social interactions and feeding schedules can also act as zeitgebers

## References

[1] Wright HK. 1901 The malarial fevers of british malaya. Singapore, China: Kelly & Walsh. See https://archive.org/details/b29000841/page/n3/mode/2up.

[2] Stangherlin A *et al*. 2021 Compensatory ion transport buffers daily protein rhythms to regulate osmotic balance and cellular physiology. Nat. Commun. **12**, 6035. (10.1038/s41467-021-25942-4) 34654800 PMC8520019

[3] Wong DCS *et al*. 2022 CRYPTOCHROMES promote daily protein homeostasis. EMBO J. **41**, e108883. (10.15252/embj.2021108883) 34842284 PMC8724739

[4] Wyse C, O’Malley G, Coogan AN, McConkey S, Smith DJ. 2021 Seasonal and daytime variation in multiple immune parameters in humans: evidence from 329,261 participants of the UK biobank cohort. iScience **24**, 102255. (10.1016/j.isci.2021.102255) 33817568 PMC8010467

[5] Collins EJ, Cervantes-Silva MP, Timmons GA, O’Siorain JR, Curtis AM, Hurley JM. 2021 Post-transcriptional circadian regulation in macrophages organizes temporally distinct immunometabolic states. Genome Res. **31**, 171–185. (10.1101/gr.263814.120) 33436377 PMC7849412

[6] Baxter M, Ray DW. 2020 Circadian rhythms in innate immunity and stress responses. Immunology **161**, 261–267. (10.1111/imm.13166) 31820826 PMC7692257

[7] Butt GR, Qayyum ZA, Jones MA. 2020 Plant defence mechanisms are modulated by the circadian system. Biology **9**, 1–11. (10.3390/biology9120454) PMC7763185 33317013

[8] Downton P, Early JO, Gibbs JE. 2020 Circadian rhythms in adaptive immunity. Immunology **161**, 268–277. (10.1111/imm.13167) 31837013 PMC7692252

[9] Duffield GE. 2024 Circadian and daily rhythms of disease vector mosquitoes. Curr. Opin. Insect Sci. **63**, 101179. (10.1016/j.cois.2024.101179) 38395256 PMC11708107

[10] Lu H, McClung CR, Zhang C. 2017 Tick tock: circadian regulation of plant innate immunity. Annu. Rev. Phytopathol. **55**, 287–311. (10.1146/annurev-phyto-080516-035451) 28590878

[11] Parsons R, Parsons R, Garner N, Oster H, Rawashdeh O. 2020 CircaCompare: a method to estimate and statistically support differences in mesor, amplitude and phase, between circadian rhythms. Bioinformatics **36**, 1208–1212. (10.1093/bioinformatics/btz730) 31588519

[12] Scheiermann C, Gibbs J, Ince L, Loudon A. 2018 Clocking in to immunity. Nat. Rev. Immunol. **18**, 423–437. (10.1038/s41577-018-0008-4) 29662121

[13] Borrmann H, Rijo-Ferreira F. 2024 Crosstalk between circadian clocks and pathogen niche. PLoS Pathog. **20**, e1012157. (10.1371/journal.ppat.1012157) 38723104 PMC11081299

[14] Prior KF, Rijo-Ferreira F, Assis PA, Hirako IC, Weaver DR, Gazzinelli RT, Reece SE. 2020 Periodic parasites and daily host rhythms. Cell Host Microbe **27**, 176–187. (10.1016/j.chom.2020.01.005) 32053788 PMC7137616

[15] Zhuang X, Edgar RS, McKeating JA. 2022 The role of circadian clock pathways in viral replication. Semin. Immunopathol. **44**, 175–182. (10.1007/s00281-021-00908-2) 35192001 PMC8861990

[16] Costantini C, Brancorsini S, Grignani F, Romani L, Bellet MM. 2025 Circadian metabolic adaptations to infections. Phil. Trans. R. Soc. B **380**, 20230473. (10.1098/rstb.2023.0473) PMC11753887 39842481

[17] Hirako IC, Ramalho T, Gazzinelli R. 2024 Immune regulation of host energy metabolism and periodicity of malaria parasites. Phil. Trans. R. Soc. B **380**, 20230511. (10.1098/rstb.2023.0511) PMC11753876 39842477

[18] Major-Styles CT, Munns J, Zeng A, Vanden Oever M, O’Neill JS, Edgar RS. 2024 Chronic cryptochrome deficiency enhances cell-intrinsic antiviral defences. Phil. Trans. R. Soc. B **380**, 20230344. (10.1098/rstb.2023.0344) PMC11753882 39842480

[19] Mihut A, O’Neill JS, Partch C, Crosby P. 2025 PERspectives on circadian cell biology. Phil. Trans. R. Soc. B **380**, 20230483. (10.1098/rstb.2023.0483) PMC11753889 39842483

[20] Munns J *et al*. 2025 Development of compounds for targeted degradation of mammalian cryptochrome proteins. Phil. Trans. R. Soc. B **380**, 20230342. (10.1098/rstb.2023.0342) PMC11753880 39842482

[21] Rageema J, Odendaal J, Ingle R, Roden LC. 2025 The role of the jasmonate signalling transcription factors MYC2/3/4 in circadian clock-mediated regulation of immunity in Arabidopsis. Phil. Trans. R. Soc. B **380**, 20230338. (10.1098/rstb.2023.0338) PMC11753874 39842487

[22] Paajanen P, Kimmey JM, Dodd AN. 2025 Circadian gating: concepts, processes, and opportunities. Phil. Trans. R. Soc. B **380**, 20230346. (10.1098/rstb.2023.0346) PMC11753883 39842478

[23] Holland JG, O’Donnell AJ, Herbert-Mainero A, Reece SE. 2025 Phenotypic and fitness consequences of plasticity in the rhythmic replication of malaria parasites. Phil. Trans. R. Soc. B **380**, 20230340. (10.1098/rstb.2023.0340) PMC11753878 39842485

[24] Larrondo LF. 2025 Circadian rhythms: pervasive, and often times evasive. Phil. Trans. R. Soc. B. **380**, 20230477. (10.1098/rstb.2023.0477) PMC12365789 39842475

[25] Rund SSC, O’Donnell AJ, Prior KF, van der Veen DR. 2025 Seasonal plasticity in daily timing of flight activity in Anopheles stephensi is driven by temperature modulation of dawn entrainment. Phil. Trans. R. Soc. B **380**, 20230343. (10.1098/rstb.2023.0343) PMC11753879 39842479

[26] Sayantan S, Tiwari J, Malik S, Stevenson T. 2025 Endocrine and molecular regulation of seasonal avian immune function. Phil. Trans. R. Soc. B **380**, 20230507. (10.1098/rstb.2023.0507) PMC11753886 39842486

[27] Greischar MA, Childs LM. 2025 Developmental synchrony and extraordinary multiplication rates in pathogenic organisms. Phil. Trans. R. Soc. B **380**, 20230337. (10.1098/rstb.2023.0337) PMC11753877 39842490

[28] Reece SE, Prior KF, Mideo N. 2017 The life and times of parasites: rhythms in strategies for within-host survival and between-host transmission. J. Biol. Rhythms **32**, 516–533. (10.1177/0748730417718904) 28845736 PMC5734377

[29] Westwood ML, O’Donnell AJ, de Bekker C, Lively CM, Zuk M, Reece SE. 2019 The evolutionary ecology of circadian rhythms in infection. Nat. Ecol. Evol. **3**, 552–560. (10.1038/s41559-019-0831-4) 30886375 PMC7614806

[30] Rees H *et al*. 2025 BioClocks UK: driving robust cycles of discovery to impact. Phil. Trans. R. Soc. B **380**, 20230345. (10.1098/rstb.2023.0345) PMC11753888 39842476

[31] Martinez-Albert E, Bless JJ, Besedovsky L. 2025 Individual associations of self-reported sleep duration, sleep quality, chronotype and social jet lag with infectious disease risk. Phil. Trans. R. Soc. B **380**, 20230472. (10.1098/rstb.2023.0472) PMC11753884 39842484

[32] Peng Z, Liu Y, Ma H, Xiao S, Au-Yeung A, Zhang L, Zeng Q, Guo Y. 2025 Characterization of extracellular vesicles released from Prochlorococcus MED4 at the steady state and under a light–dark cycle. Phil. Trans. R. Soc. B **380**, 20230339. (10.1098/rstb.2023.0339) PMC11753881 39842488

[33] Edgar RS, Stangherlin A, Nagy AD, Nicoll MP, Efstathiou S, O’Neill JS, Reddy AB. 2016 Cell autonomous regulation of herpes and influenza virus infection by the circadian clock. Proc. Natl Acad. Sci. USA **113**, 10085–10090. (10.1073/pnas.1601895113) 27528682 PMC5018795

[34] O’Donnell AJ, Schneider P, McWatters HG, Reece SE. 2011 Fitness costs of disrupting circadian rhythms in malaria parasites. Proc. R. Soc. B. **278**, 2429–2436. (10.1098/rspb.2010.2457) PMC3125626 21208950

[35] Kiessling S, Dubeau-Laramée G, Ohm H, Labrecque N, Olivier M, Cermakian N. 2017 The circadian clock in immune cells controls the magnitude of Leishmania parasite infection. Sci. Rep. **7**, 10892. (10.1038/s41598-017-11297-8) 28883509 PMC5589941

[36] Sengupta S *et al*. 2019 Circadian control of lung inflammation in influenza infection. Nat. Commun. **10**, 4107. (10.1038/s41467-019-11400-9) 31511530 PMC6739310

[37] Zhuang X, Lai AG, McKeating JA, Rowe I, Balfe P. 2018 Daytime variation in hepatitis C virus replication kinetics following liver transplant. Wellcome Open Res. **3**, 96. (10.12688/wellcomeopenres.14696.2) 30175249 PMC6107978

[38] Feigin RD, Joaquin VHS, Haymond MW, Wyatt RG. 1969 Daily periodicity of susceptibility of mice to pneumococcal infection. Nature **224**, 379–380. (10.1038/224379a0) 5343888

[39] Zhuang X, Wang W, Borrmann H, Balfe P, Matthews PC, Eyre DW, Klerman EB, McKeating JA. 2022 Time-of-day variation in SARS-CoV-2 RNA levels during the second wave of COVID-19. Viruses **14**, 1728. (10.3390/v14081728) 36016350 PMC9413669

[40] Kawaguchi Y, Tanaka M, Yokoymama A, Matsuda G, Kato K, Kagawa H, Hirai K, Roizman B. 2001 Herpes simplex virus 1α regulatory protein ICP0 functionally interacts with cellular transcription factor BMAL1. Proc. Natl Acad. Sci. USA **98**, 1877–1882. (10.1073/pnas.041592598) 11172044 PMC29350

[41] Mukherji A *et al*. 2024 An atlas of the human liver diurnal transcriptome and its perturbation by hepatitis C virus infection. Nat. Commun. **15**, 7486. (10.1038/s41467-024-51698-8) 39209804 PMC11362569

[42] Pearson JA, Wong FS, Wen L. 2020 Crosstalk between circadian rhythms and the microbiota. Immunology **161**, 278–290. (10.1111/imm.13278) 33090484 PMC7692254

[43] Man K, Loudon A, Chawla A. 2016 Immunity around the clock. Science **354**, 999–1003. (10.1126/science.aah4966) 27885005 PMC5247264

[44] Dunlap JC, Loros JJ, DeCoursey PJ. 2004 Chronobiology: biological timekeeping. Sunderland, MA: Sinauer Associates.

[45] Pittendrigh CS. 1960 Circadian rhythms and the circadian organization of living systems. Cold Spring Harb. Symp. Quant. Biol. **25**, 159–184. (10.1101/sqb.1960.025.01.015) 13736116

[46] Lück S, Thurley K, Thaben PF, Westermark PO. 2014 Rhythmic degradation explains and unifies circadian transcriptome and proteome data. Cell Rep. **9**, 741–751. (10.1016/j.celrep.2014.09.021) 25373909

[47] Stangherlin A, Seinkmane E, O’Neill JS. 2021 Understanding circadian regulation of mammalian cell function, protein homeostasis, and metabolism. Curr. Opin. Syst. Biol. **28**, e100391. (10.1016/j.coisb.2021.100391) PMC8660647 34950808

[48] Crosby P, Partch CL. 2020 New insights into non-transcriptional regulation of mammalian core clock proteins. J. Cell. Sci. **133**, jcs241174. (10.1242/jcs.241174) 32934011 PMC7520459

[49] Edgar RS *et al*. 2012 Peroxiredoxins are conserved markers of circadian rhythms. Nature **485**, 459–464. (10.1038/nature11088) 22622569 PMC3398137

[50] Lipton JO *et al*. 2015 The circadian protein BMAL1 regulates translation in response to S6K1-mediated phosphorylation. Cell **161**, 1138–1151. (10.1016/j.cell.2015.04.002) 25981667 PMC4447213

[51] O’Neill JS, van Ooijen G, Dixon LE, Troein C, Corellou F, Bouget FY, Reddy AB, Millar AJ. 2011 Circadian rhythms persist without transcription in a eukaryote. Nature **469**, 554–558. (10.1038/nature09654) 21270895 PMC3040569

[52] O’Neill J, Reddy A. 2011 Circadian clocks in human red blood cells. Nature New Biol. **469**, 498–503. (10.1038/nature09702) PMC3040566 21270888

[53] Scialdone A, Howard M. 2015 How plants manage food reserves at night: quantitative models and open questions. Front. Plant Sci. **6**, 204. (10.3389/fpls.2015.00204) 25873925 PMC4379750

[54] Tomita J, Nakajima M, Kondo T, Iwasaki H. 2005 No transcription-translation feedback in circadian rhythm of KaiC phosphorylation. Science **307**, 251–254. (10.1126/science.1102540) 15550625

[55] Eskandari R, Ratnayake L, Lakin-Thomas PL. 2021 Shared components of the FRQ-less oscillator and tor pathway maintain rhythmicity in Neurospora J. Biol. Rhythms **36**, 329–345. (10.1177/0748730421999948) 33825541 PMC8276340

[56] Jabbur ML, Dani C, Spoelstra K, Dodd AN, Johnson CH. 2024 Evaluating the adaptive fitness of circadian clocks and their evolution. J. Biol. Rhythms **39**, 115–134. (10.1177/07487304231219206) 38185853 PMC10994774

[57] Sharma VK. 2003 Adaptive significance of circadian clocks. Chronobiol. Int. **20**, 901–919. (10.1081/cbi-120026099) 14680135

[58] Graf A, Schlereth A, Stitt M, Smith AM. 2010 Circadian control of carbohydrate availability for growth in Arabidopsis plants at night. Proc. Natl Acad. Sci. USA **107**, 9458–9463. (10.1073/pnas.0914299107) 20439704 PMC2889127

[59] Ouyang Y, Andersson CR, Kondo T, Golden SS, Johnson CH. 1998 Resonating circadian clocks enhance fitness in cyanobacteria. Proc. Natl Acad. Sci. USA **95**, 8660–8664. (10.1073/pnas.95.15.8660) 9671734 PMC21132

[60] Dodd AN, Salathia N, Hall A, Kévei E, Tóth R, Nagy F, Hibberd JM, Millar AJ, Webb AAR. 2005 Plant circadian clocks increase photosynthesis, growth, survival, and competitive advantage. Science **309**, 630–633. (10.1126/science.1115581) 16040710

[61] Spoelstra K, Wikelski M, Daan S, Loudon ASI, Hau M. 2016 Natural selection against a circadian clock gene mutation in mice. Proc. Natl Acad. Sci. USA **113**, 686–691. (10.1073/pnas.1516442113) 26715747 PMC4725470

[62] Bloch G, Bar-Shai N, Cytter Y, Green R. 2017 Time is honey: circadian clocks of bees and flowers and how their interactions may influence ecological communities. Phil. Trans. R. Soc. B **372**, 20160256. (10.1098/rstb.2016.0256) 28993499 PMC5647282

[63] Jabbur ML, Bratton BP, Johnson CH. 2024 Bacteria can anticipate the seasons. Science **385**, 1105. (10.1126/science.ado8588) 39236161 PMC11473183

[64] Mendaña A *et al*. 2024 Long-term evolution reveals the role of the circadian cycle in the environmental adaptation of cyanobacteria. bioRxiv 2024.03.12.584591. (10.1101/2024.03.12.584591)

[65] Kalamvoki M, Roizman B. 2010 Circadian CLOCK histone acetyl transferase localizes at ND10 nuclear bodies and enables herpes simplex virus gene expression. Proc. Natl Acad. Sci. USA **107**, 17721–17726. (10.1073/pnas.1012991107) 20876123 PMC2955081

[66] de Bekker C, Das B. 2022 Hijacking time: how Ophiocordyceps fungi could be using ant host clocks to manipulate behavior. Parasite Immunol. **44**, e12909. (10.1111/pim.12909) 35103986 PMC9287076

[67] Schneider P, Rund SSC, Smith NL, Prior KF, O’Donnell AJ, Reece SE. 2018 Adaptive periodicity in the infectivity of malaria gametocytes to mosquitoes. Proc. R. Soc. B **285**, 20181876. (10.1098/rspb.2018.1876) PMC6191691 30282657

[68] Boy-Waxman S, Olivier M, Cermakian N. 2024 Clockwork intruders: do parasites manipulate their hosts’ circadian rhythms? Curr. Res. Parasitol. Vector Borne Dis. **5**, 100171. (10.1016/j.crpvbd.2024.100171) 38545439 PMC10966150

[69] Ehlers A *et al*. 2018 BMAL1 links the circadian clock to viral airway pathology and asthma phenotypes. Mucosal Immunol. **11**, 97–111. (10.1038/mi.2017.24) 28401936 PMC5638664

[70] Sundar IK, Ahmad T, Yao H, Hwang J-woong, Gerloff J, Lawrence BP, Sellix MT, Rahman I. 2015 Influenza A virus-dependent remodeling of pulmonary clock function in a mouse model of COPD. Sci. Rep. **4**, 9927. (10.1038/srep09927) 25923474 PMC4413879

[71] Huitron-Resendiz S, Marcondes MCG, Flynn CT, Lanigan CMS, Fox HS. 2007 Effects of simian immunodeficiency virus on the circadian rhythms of body temperature and gross locomotor activity. Proc. Natl Acad. Sci. USA **104**, 15138–15143. (10.1073/pnas.0707171104) 17846423 PMC1986626

[72] Clark JP, Sampair CS, Kofuji P, Nath A, Ding JM. 2005 HIV protein, transactivator of transcription, alters circadian rhythms through the light entrainment pathway. Am. J. Physiol. Regul. Integr. Comp. Physiol. **289**, R656–R662. (10.1152/ajpregu.00179.2005) 15860648

[73] Prior KF, O’Donnell AJ, Rund SSC, Savill NJ, van der Veen DR, Reece SE. 2019 Host circadian rhythms are disrupted during malaria infection in parasite genotype-specific manners. Sci. Rep. **9**, 10905. (10.1038/s41598-019-47191-8) 31358780 PMC6662749

[74] Wang T *et al*. 2014 HIV Tat protein affects circadian rhythmicity by interfering with the circadian system. HIV Med. **15**, 565–570. (10.1111/hiv.12154) 24750691 PMC4285855

[75] Zhuang X *et al*. 2021 Circadian control of hepatitis B virus replication. Nat. Commun. **12**, 1658. (10.1038/s41467-021-21821-0) 33712578 PMC7955118

[76] Zhuang X *et al*. 2019 The circadian clock components BMAL1 and REV-ERBα regulate flavivirus replication. Nat. Commun. **10**, 377. (10.1038/s41467-019-08299-7) 30670689 PMC6343007

[77] Rijo-Ferreira F, Acosta-Rodriguez VA, Abel JH, Kornblum I, Bento I, Kilaru G, Klerman EB, Mota MM, Takahashi JS. 2020 The malaria parasite has an intrinsic clock. Science **368**, 746–753. (10.1126/science.aba2658) 32409471 PMC7409452

[78] Rijo-Ferreira F, Pinto-Neves D, Barbosa-Morais NL, Takahashi JS, Figueiredo LM. 2017 Trypanosoma brucei metabolism is under circadian control. Nat. Microbiol. **2**, 17032. (10.1038/nmicrobiol.2017.32) 28288095 PMC5398093

[79] Henríquez-Urrutia M *et al*. 2022 Circadian oscillations in Trichoderma atroviride and the role of core clock components in secondary metabolism, development, and mycoparasitism against the phytopathogen Botrytis cinerea. eLife **11**, e71358. (10.7554/eLife.71358) 35950750 PMC9427114

[80] Hevia MA, Canessa P, Müller-Esparza H, Larrondo LF. 2015 A circadian oscillator in the fungus Botrytis cinerea regulates virulence when infecting Arabidopsis thaliana. Proc. Natl Acad. Sci. USA **112**, 8744–8749. (10.1073/pnas.1508432112) 26124115 PMC4507220

[81] Mideo N, Reece SE, Smith AL, Metcalf CJE. 2013 The Cinderella syndrome: why do malaria-infected cells burst at midnight? Trends Parasitol. **29**, 10–16. (10.1016/j.pt.2012.10.006) 23253515 PMC3925801

[82] Holland JG, Prior KF, O’Donnell AJ, Reece SE. 2024 Testing the evolutionary drivers of malaria parasite rhythms and their consequences for host–parasite interactions. Evol. Appl. **17**, e13752. (10.1111/eva.13752) 39006006 PMC11246599

[83] Hughes ME *et al*. 2017 Guidelines for genome-scale analysis of biological rhythms. J. Biol. Rhythms **32**, 380–393. (10.1177/0748730417728663) 29098954 PMC5692188

[84] Laloum D, Robinson-Rechavi M. 2020 Methods detecting rhythmic gene expression are biologically relevant only for strong signal. PLoS Comput. Biol. **16**, e1007666. (10.1371/journal.pcbi.1007666) 32182235 PMC7100990

[85] Hughes ME, Hogenesch JB, Kornacker K. 2010 JTK_CYCLE: an efficient nonparametric algorithm for detecting rhythmic components in genome-scale data sets. J. Biol. Rhythms **25**, 372–380. (10.1177/0748730410379711) 20876817 PMC3119870

[86] Yang R, Su Z. 2010 Analyzing circadian expression data by harmonic regression based on autoregressive spectral estimation. Bioinformatics **26**, i168–74. (10.1093/bioinformatics/btq189) 20529902 PMC2881374

[87] Thaben PF, Westermark PO. 2014 Detecting rhythms in time series with RAIN. J. Biol. Rhythms **29**, 391–400. (10.1177/0748730414553029) 25326247 PMC4266694

[88] Greischar MA, Savill NJ, Reece SE, Mideo N. 2024 How to quantify developmental synchrony in malaria parasites. Front. Malar. **2**, 2. (10.3389/fmala.2024.1386266)

[89] Greischar MA, Reece SE, Savill NJ, Mideo N. 2019 The challenge of quantifying synchrony in malaria parasites. Trends Parasitol. **35**, 341–355. (10.1016/j.pt.2019.03.002) 30952484

[90] Straume M. 2004 DNA microarray time series analysis: automated statistical assessment of circadian rhythms in gene expression patterning. Meth. Enzymol. **383**, 149–166. (10.1016/S0076-6879(04)83007-6) 15063650

[91] Zieliński T, Hay J, Millar AJ. 2022 Period estimation and rhythm detection in timeseries data using BioDare2, the free, online, community resource. Methods Mol. Biol. **2398**, 15–32. (10.1007/978-1-0716-1912-4_2) 34674164

[92] ClockLab. Actimetrics. See https://actimetrics.com/products/clocklab/ (accessed 12 September 2024).

[93] Cenek L, Klindziuk L, Lopez C, McCartney E, Martin Burgos B, Tir S, Harrington ME, Leise TL. 2020 CIRCADA: Shiny Apps for Exploration of Experimental and Synthetic Circadian Time Series with an Educational Emphasis. J. Biol. Rhythms **35**, 214–222. (10.1177/0748730419900866) 31986956 PMC7752169

[94] Exploring Circadian Time Series. 2024 Retrieved 3 September 2024. See https://sites.smith.edu/circada/.

[95] Geissmann Q, Garcia Rodriguez L, Beckwith EJ, Gilestro GF. 2019 Rethomics: an R framework to analyse high-throughput behavioural data. PLoS One **14**, e0209331. (10.1371/journal.pone.0209331) 30650089 PMC6334930

[96] Mönke G, Sorgenfrei FA, Schmal C, Granada AE. 2020 Optimal time frequency analysis for biological data - pyBOAT. BioRxiv. (10.1101/2020.04.29.067744)

[97] Wu G, Anafi RC, Hughes ME, Kornacker K, Hogenesch JB. 2016 MetaCycle: an integrated R package to evaluate periodicity in large scale data. Bioinformatics **32**, 3351–3353. (10.1093/bioinformatics/btw405) 27378304 PMC5079475

[98] Zielinski T, Moore AM, Troup E, Halliday KJ, Millar AJ. 2014 Strengths and limitations of period estimation methods for circadian data. PLoS One **9**, e96462. (10.1371/journal.pone.0096462) 24809473 PMC4014635

[99] Mulder K, Klugkist I. 2017 Bayesian estimation and hypothesis tests for a circular generalized linear model. J. Math. Psychol. **80**, 4–14. (10.1016/j.jmp.2017.07.001)

[100] Singer JM, Hughey JJ. 2019 LimoRhyde: a flexible approach for differential analysis of rhythmic transcriptome data. J. Biol. Rhythms **34**, 5–18. (10.1177/0748730418813785) 30472909 PMC6376636

[101] Ruan W, Yuan X, Eltzschig HK. 2021 Circadian rhythm as a therapeutic target. Nat. Rev. Drug Discov. **20**, 287–307. (10.1038/s41573-020-00109-w) 33589815 PMC8525418

[102] Alibhai FJ, Tsimakouridze EV, Chinnappareddy N, Wright DC, Billia F, O’Sullivan ML, Pyle WG, Sole MJ, Martino TA. 2014 Short-term disruption of diurnal rhythms after murine myocardial infarction adversely affects long-term myocardial structure and function. Circ. Res. **114**, 1713–1722. (10.1161/CIRCRESAHA.114.302995) 24687134

[103] Farag HI *et al*. 2024 One Health: circadian medicine benefits both non-human animals and humans alike. J. Biol. Rhythms **39**, 237–269. (10.1177/07487304241228021) 38379166 PMC11141112

[104] Zhang R, Lahens NF, Ballance HI, Hughes ME, Hogenesch JB. 2014 A circadian gene expression atlas in mammals: implications for biology and medicine. Proc. Natl Acad. Sci. USA **111**, 16219–16224. (10.1073/pnas.1408886111) 25349387 PMC4234565

[105] Cermakian N, Stegeman SK, Tekade K, Labrecque N. 2022 Circadian rhythms in adaptive immunity and vaccination. Semin. Immunopathol. **44**, 193–207. (10.1007/s00281-021-00903-7) 34825270

[106] Schneider P, Reece SE. 2021 The private life of malaria parasites: strategies for sexual reproduction. Mol. Biochem. Parasitol. **244**, 111375. (10.1016/j.molbiopara.2021.111375) 34023299 PMC8346949

[107] Teuscher F, Gatton ML, Chen N, Peters J, Kyle DE, Cheng Q. 2010 Artemisinin‐induced dormancy in Plasmodium falciparum: duration, recovery rates, and implications in treatment failure. J. Infect. Dis. **202**, 1362–1368. (10.1086/656476) 20863228 PMC2949454

